# A Ubiquitin Shuttle DC-UbP/UBTD2 Reconciles Protein Ubiquitination and Deubiquitination via Linking UbE1 and USP5 Enzymes

**DOI:** 10.1371/journal.pone.0107509

**Published:** 2014-09-10

**Authors:** Ai-Xin Song, Hui Yang, Yong-Guang Gao, Chen-Jie Zhou, Yu-Hang Zhang, Hong-Yu Hu

**Affiliations:** State Key Laboratory of Molecular Biology, Institute of Biochemistry and Cell Biology, Shanghai Institutes for Biological Sciences, Chinese Academy of Sciences, Shanghai, China; Loyola University Chicago, Stritch School of Medicine, United States of America

## Abstract

The ubiquitination levels of protein substrates in eukaryotic cells are delicately orchestrated by various protein cofactors and enzymes. Dendritic cell-derived ubiquitin (Ub)-like protein (DC-UbP), also named as Ub domain-containing protein 2 (UBTD2), is a potential Ub shuttle protein comprised of a Ub-like (UbL) domain and a Ub-binding domain (UBD), but its biological function remains largely unknown. We identified two Ub-related enzymes, the deubiquitinating enzyme USP5 and the Ub-activating enzyme UbE1, as interacting partners of DC-UbP from HEK 293T cells. Biochemical studies revealed that the tandem UBA domains of USP5 and the C-terminal Ub-fold domain (UFD) of UbE1 directly interacted with the C-terminal UbL domain of DC-UbP but on the distinct surfaces. Overexpression of DC-UbP in HEK 293T cells enhanced the association of these two enzymes and thus prompted cellular ubiquitination, whereas knockdown of the protein reduced the cellular ubiquitination level. Together, DC-UbP may integrate the functions of USP5 and UbE1 through interacting with them, and thus reconcile the cellular ubiquitination and deubiquitination processes.

## Introduction

Ubiquitination is one of the common post-translational modifications of proteins in eukaryotic organisms [1]. By working as a versatile regulatory signal controlling protein stability, cellular localization and biological function, ubiquitination plays very important roles in gene regulation, cell cycle, cellular protein level, cell signaling and so on [2], [3], [4]. In these processes, ubiquitin is covalently attached to a target protein with the cascade participation of three enzymes, Ub-activating enzyme E1 (UbE1), Ub-conjugating enzyme E2 (UbE2) and Ub E3 ligase (UbE3) [1], [5]. UbE1 (Uba1 in yeast) is a unique enzyme that universally activates Ub molecules for conjugating to a UbE2 and transferring to substrates assisted by one of the numerous UbE3 ligases.

Ubiquitination is regulated in multi-levels and aspects, and most importantly, this process can be reversed by deubiquitinating enzymes (DUB). DUB selectively removes Ub or edits the length or type of Ub chain on substrate [6]. There are five families of DUBs in eukaryotes, which may have different locations, targets or mechanisms, and their activities and specificities on substrates are extremely diverse [7], [8]. The largest group ubiquitin-specific protease (USP) contains a catalytic domain usually consisting of a Cys box and a His box [9]. USP typically cleaves Ub conjugates from ubiquitinated protein substrates or unanchored Ub chains.

It is generally considered that the ubiquitination levels of protein substrates in cells are highly orchestrated with various protein cofactors [10], [11], including shuttle factors like Rad23 and Dsk2. Dendritic cell-derived ubiquitin-like protein (DC-UbP), also named as Ub domain-containing protein 2 (UBTD2), is a novel Ub domain-containing protein firstly identified in dendritic cells and implicated in ubiquitination pathway [12]. Our previous work has elucidated the solution structure of the C-terminal part of DC-UbP (UbP_C), indicating that UbP_C is structurally comprised of a typical Ub-like (UbL) fold but lacks the conserved diglycine tail necessary to Ub modification [13]. The UbL structure also displays a positively-charged surface distinct from Ub molecule, suggesting that the UbL domain of DC-UbP may have its unique interacting partner and cellular function. We also solved the novel structure of the N-terminal part of DC-UbP (UbP_N) and found that it is potentially a Ub-binding domain (UBD) [14]. More importantly, the DC-UbP protein is a combination of UbL and UBD domains, which increase the possibility for DC-UbP to be involved in the ubiquitination process or other relevant pathways [15]. However, the detailed biological function of DC-UbP and its underlying mechanism are still unclear.

To unravel the biological function of DC-UbP in protein ubiquitination and delivery of ubiquitinated substrates, we firstly performed pull-down experiments to characterize its potential interacting partners that led to identify two enzymes, UbE1 and USP5, which function cooperatively in protein ubiquitination and deubiquitination. Then we confirmed their interactions *in* *vitro* and in cell model by biochemical methods. DC-UbP may play a role in mediating association of UbE1 and USP5 and thus modulating the ubiquitination levels of protein substrates in cells. Finally, a schematic model is proposed that DC-UbP participates in the delicate regulation of cellular ubiquitination and deubiquitination processes through linking the UbE1 and USP5 enzymes.

## Materials and Methods

### Plasmids, antibodies and reagents

PCR-amplified cDNAs of human DC-UbP and its N- and C-terminal domains (residues 14–141, 129–234) were cloned into the vector pET22b(+), pGEX-4T-3 or pCMV-tag2B, respectively. UbE1 was cloned from mouse cDNA library and ligated into pET28a vector (Invitrogen) for purification. The FH (1–439) and SCCH (622–891) fragments of UbE1 were generated by PCR amplification and inserted into pET22b(+) vector. To get stable expression, the UFD domain of UbE1 (950–1058) was cloned into pGEX-4T-3 vector at the *BamH* I and *Xho* I sites. The cDNA encoding human USP5 was cloned from the cDNA library of HEK 293T cells; then it was ligated into pET-MG vector (*BamH* I/*Xho* I) for expression in *E. coli* or pcDNA3.1-Myc/His vector (*BamH* I/*Xho* I) for expression in mammalian cells. The cDNAs encoding the UBA12 (residues 625–749), UBA1 (631–692) and ZnF (169–289) domains of USP5 were generated by PCR amplification and inserted into pGBTNH or pET-M vector at the *BamH* I and *Xho* I sites, respectively. All constructs were verified by DNA sequencing.

The following antibodies were used: mouse anti-FLAG (Sigma), rabbit anti-FLAG (Sigma), mouse anti-Myc (Cell Signaling), mouse anti-UbE1 (Abcam), rabbit anti-USP5 (Proteintech), mouse anti-DC-UbP (Sigma), mouse anti-Ub (Santa Cruz), anti-His tag and mouse anti-GAPDH (Sigma). Ni-NTA (Qiagen) and glutathione-agarose beads (Amersham Biosciences) were utilized for protein purification or GST pull-down experiments. Protein A beads were the product of GE Healthcare and the anti-FLAG M2-Agarose were the product of Sigma. ^15^N-NH_4_Cl and D_2_O were purchased from Cambridge Isotope Laboratories; the Ub-AMC substrate was from Boston Biochem. Other reagents used were ECL detection reagents (Pierce), Polyjet (SignaGen), and Lipofectamine 2000 transfection reagent (Invitrogen).

### Pull-down with cell lysates and LC-MS/MS analysis

GST and GST-fused DC-UbP were expressed in *E. coli* and purified with glutathione-agarose column. HEK 293T cells (American Type Culture Collection) were cultured at 37°C in Dulbecco’s modified Eagle’s medium (DMEM, Gibco) supplemented with 10% (V/V) fetal bovine serum (Gibco), penicillin and streptomycin, and under a humidified atmosphere containing 5% CO_2_. The cells of 20×10 cm plates were harvested and re-suspended in the lysis Buffer A (20 mM HEPES, 150 mM NaCl, 1 mM EDTA, 10% glycerol, 1 mM DTT, 1% Triton X-100, pH 7.4) with 1 mM PMSF and Roche Protease Cocktail (1%). After incubating on ice for 15 min, the cell lysates were centrifuged for 10 min at 10,000 rpm and the clear supernatants were loaded onto the glutathione-agarose column (500 µL beads for each) pre-saturated with GST or GST-DC-UbP. After incubation for 3 h or overnight, the proteins were washed with the lysis buffer containing 350 mM NaCl and then eluted by a GSH elution buffer (50 mM Tris-HCl, 10 mM GSH, pH 8.0). The elutes were precipitated with 20% TCA, and the pellets were re-suspended with the loading buffer in 1/10 of the original volume for SDS-PAGE or silver staining. Different bands were selected and cut from the gel, washed and digested for 20 h by trypsin, and then the peptides were separated and detected by liquid chromatography (LC) coupled MS/MS analysis (LTQ, Thermo Finnigan; Agilent Technologies). The MS data were processed by using BIOWORKS software to give a peak list files, and the HUMAN (Version3.36) database was used for protein searching.

### GST pull-down assay

The concentrations of the proteins were determined spectrophotometrically at 280 nm with the extinction coefficient of each protein. For GST pull-down assay, the purified GST or GST-fused UFD was mixed with glutathione-agarose beads (30 µL) for 1 h at 4°C. Then His-tagged DC-UbP or UbP_C was added and the mixtures were incubated for another 6 h at 4°C. The beads were washed three times with PBS buffer, and the proteins were eluted by GSH. The eluted proteins were subjected to SDS-PAGE (15% gel) and analyzed by immunoblotting with anti-His antibody. Proteins were also detected by Coomassie blue staining.

### Transfection, immunoprecipitation and Western blotting

FLAG tagged DC-UbP or its fragments were co-transfected with Myc-USP5 into HEK 293T cells (Polyjet reagent) and cultured for another 36 to 48 h. The cell lysates were centrifuged and then the supernatants were subjected to SDS-PAGE. For immunoprecipitation, the clear supernatants were incubated with equal amount of monoclonal FLAG antibody or Myc antibody for above 3 h at 4°C. The bound beads were washed three times with the lysis buffer and once with PBS buffer, and then the proteins were released from the beads by glycine-HCl buffer (100 mM, pH 3.5). The samples were fractionated by SDS-PAGE and subjected to immunoblotting for USP5, DC-UbP and Ub, respectively. Endogenous UbE1 was detected directly by anti-UbE1 antibody. Co-transfection of pCMV-tag2B vector with Myc-USP5 was set as a control.

### Isotope labeling of proteins and NMR titration

The ^15^N-labeled DC-UbP, UbP_N and UbP_C proteins were prepared by using the M9 minimal medium containing ^15^N-NH_4_Cl, and were purified by Ni^+^-NTA affinity column. The proteins were further purified via Superdex-75 column (GE Healthcare) and then desalted and lyophilized. For NMR titration, the samples (100 µM) were dissolved in a phosphate buffer (20 mM phosphate, 50 mM NaCl, pH 6.5) containing 10% D_2_O. All NMR spectra were acquired at 298 K on a Bruker Avance 600-MHz NMR spectrometer equipped with a TCI cryoprobe. ^15^N-labeled protein or domain was titrated stepwise with unlabeled UbE1, USP5 or their various fragments (ZnF, UBA12 and UBA1 from USP5; FH, SCCH and UFD from UbE1). A series of ^1^H-^15^N HSQC spectra were obtained and the chemical shift changes were measured at different molar ratios. Data processing was followed as described previously [16], [17].

### *In vitro* deubiquitination assay

The deubiquitinating activity of USP5 was measured for cell lysates or the components from GST-DC-UbP pull-down by using Ub-AMC substrate as described previously [18], [19]. All the experiments were performed in a buffer of 50 mM Tris (pH 8.0) 150 mM NaCl, 10 µg/mL ovalbumin and 1 mM DTT. Ub-AMC (250 nM) was incubated with various samples for measuring the hydrolytic activities. The fluorescence of AMC release was recorded on a Fluorescence Spectrophotometer (Varian Cary Eclipse) during the reaction process with an excitation at 380 nm and the emission at 460 nm.

### *In vitro* ubiquitination assay

The cell lysates from 2×10 cm plates were incubated with GST or GST-DC-UbP for 3∼4 h, then eluted with 100 µL GSH buffer. The reactions of Ub activation and conjugation to E2 enzyme were carried out by mixing 10 µL of the pull-down components with 2 µM UbcH5C and 2 µM Ub and incubating in 20 µL of the reaction system (20 mM Tris, pH 7.5, 50 mM NaCl, 0.1 mM DTT, 4 mM ATP and 10 mM MgCl_2_). The reaction lasted for 10 min at 25°C and was terminated with the sample loading buffer (with or without DTT). The samples were then subjected to SDS-PAGE, followed by anti-Ub antibody detection. The purified UbE1 (50 nM) or whole-cell lysates (1/10) was used as a control. K48-linked diUb was prepared according to the literatures [20], [21]. The experiment for Ub conjugation to E2-25K was carried out under the similar condition, where DC-UbP (BSA as a control) was added in the reaction system for testing its potential effect on UbE1 activity and Ub conjugation.

### RNAi silencing of DC-UbP

Two siRNAs targeting DC-UbP/UBTD2 and a negative control siRNA were synthesized (Shanghai GenePharma). The siRNA sequences are 5′-GGCACAAGCAAUCAUUGAU-3′(1^#^), 5′-CGCCAAUCAACAUGAUAGA-3′(2^#^), and 5′-UUCUCCGAACGUGUCACGU-3′(Ctrl). Each siRNA (3 µg) was transfected into HEK 293T cells using lipofectamine 2000 (Invitrogen) following the manufacture’s instructions. The cells were lysed with lysis Buffer B (20 mM Tris, 50 mM NaCl, 0.5% SDS, pH 7.5) including 1 mM PMSF and Roche Protease Cocktail (1%), then the lysates were subjected to SDS-PAGE and Western blotting analysis.

## Results

### Identification of the interacting partners for DC-UbP protein

**Figure 1:**
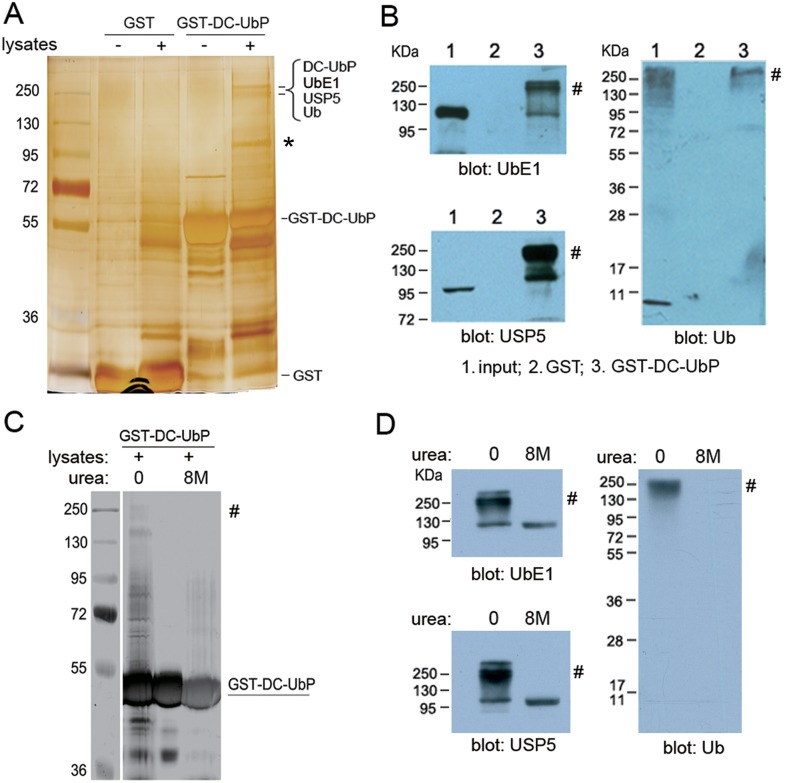
Identification of interacting partners for DC-UbP protein from HEK 293T cell lysates. **A,** SDS-PAGE analysis of the proteins pulled down by GST-DC-UbP. There exist two protein bands around 250 kDa from the protein species pulled down by GST-fused DC-UbP. By using LC-MS/MS assay, UbE1, USP5 and Ub have been identified from each band. GST was set as a control. The samples were eluted by Buffer A containing 350 mM NaCl. *, this band around 100 kDa was identified as P97/VCP. **B,** Immunoblotting detection of the identified proteins with antibodies against UbE1, USP5 or Ub. #, this high-molecular-weight species could also be detected by all the three antibodies applied. **C,** Dissociation of the high-molecular-weight species in the presence of 8 M urea. The 250-kDa bands were disappeared in the silver-staining gel. After TCA precipitation, the pellet was re-suspended with 8 M urea. **D,** Western blotting detection of the individual components in 8 M urea dissociated from the high-molecular-weight species. The antibodies against UbE1, USP5 and Ub were applied. The 120- and 95-kDa proteins were UbE1 and USP5, respectively. The smear bands for Ub derivatives could not be detected due to very small amounts in the gel.

Human DC-UbP/UBTD2 is a UbL domain-containing protein that is implicated in ubiquitination pathway [12], but its interacting partners and biological function remain largely unknown. To study its function, we firstly performed pull-down experiments on HEK 293T cell lysates with GST-fused DC-UbP protein. As compared with GST control, the sample pulled down by GST-DC-UbP exhibited mainly three additional protein bands at 100 kDa and around 250 kDa respectively in the silver staining gel (Fig. 1A). Combined with mass spectrometry (LC-MS/MS) analysis and database searches (Fig. S1), we revealed two important enzymes, ubiquitin-activating enzyme E1 (UbE1) and ubiquitin-specific protease 5 (USP5), in the bands around 250 kDa (Fig. 1A). Additionally, Ub molecules, polyUb chains or Ub precursors had also been identified together with GST-DC-UbP in these bands (Fig. S1). Interestingly, all proteins identified in the 250-kDa bands are involved in or related to the ubiquitination processes. These proteins could be detected with corresponding antibodies, and all these three antibodies displayed positive results to the high molecular-weight species (Fig. 1B), implying that DC-UbP interacts with UbE1 or USP5 and Ub plays a role in their associations. We presumed putatively that these proteins pulled down by GST-DC-UbP formed a large complex or assembly even under the SDS-PAGE condition. However, under the condition of 8 M urea, the band for this complex became very weak or almost undetectable (Fig. 1C). Note that the bands of associated proteins could also be blotted by the Ub antibody but disappeared in the presence of 8 M urea (Fig. 1D), suggesting the involvement of the Ub molecules or ubiquitinated species in the complex. Moreover, the respective bands of UbE1 (∼120 kDa) and USP5 (∼95 kDa) could also be detected with their respective antibodies in the sample treated with 8 M urea (Fig. 1D). Thus, the SDS-resistant DC-UbP complex or assembly formed at least by UbE1, USP5 and Ub is through non-covalent interactions, which is implicated in the ubiquitination and deubiquitination processes.

### DC-UbP directly interacts with UbE1 or USP5

**Figure 2:**
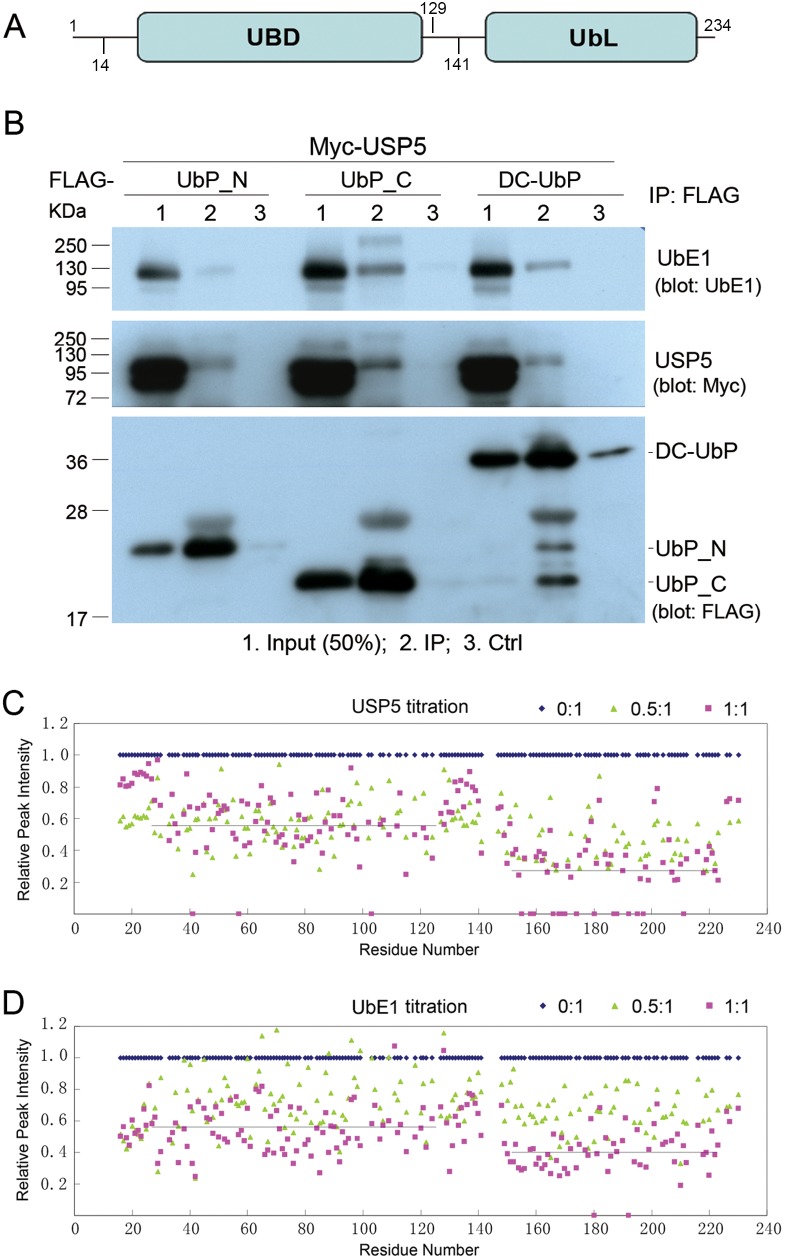
Confirmation of the interaction between DC-UbP and USP5 or UbE1. **A,** DC-UbP contains two separate domains, the N-terminal UBD and the C-terminal UbL. The N-terminal construct (UbP_N) includes residues 14–141, while the C-terminal (UbP_C) contains residues 129–234. **B,** Co-IP experiment testing the interactions between DC-UbP and USP5 or UbE1. FLAG-tagged DC-UbP, UbP_N or UbP_C was co-transfected with Myc-USP5 into HEK 293T cells. The mouse anti-FLAG M2-agarose was used for the IP experiment. Endogenous UbE1 was detected with an antibody against UbE1. Ctrl, protein-A/G beads without anti-FLAG antibody. **C,** NMR titration of DC-UbP with USP5. The relative peak intensities for both N- and C-terminal domains of DC-UbP were displayed upon addition of molar ratios of USP5 into the ^15^N-labeled DC-UbP protein. For DC-UbP alone, the peak intensities (heights) were normalized as 1 for all the peaks of DC-UbP except those for prolines and unassigned residues. The lines indicate the mean peak intensities for each domain of DC-UbP at a molar ratio of 1∶1, which are 0.56 for the UBD domain and 0.27 for the UbL domain, respectively. **D,** As in (**C**), NMR titration of DC-UbP with UbE1. The mean peak intensities are 0.54 and 0.39 for UBD and UbL, respectively, at a molar ratio of 1∶1.

To verify the interactions among DC-UbP, UbE1 and USP5, we performed co-immunoprecipitation (co-IP) experiments by co-transfecting with FLAG-DC-UbP and Myc-USP5 (Fig. 2B). The result showed that DC-UbP could immunoprecipitate USP5 as well as endogenous UbE1, that is to say, the protein interacted with USP5 or UbE1. Since DC-UbP contains two domains (Fig. 2A), we tested which domain interacts with UbE1 or USP5. The co-IP data indicated that the C-terminal part of DC-UbP (UbP_C, residues 129–234) specifically interacted with USP5 or UbE1 (Fig. 2B), whereas the N-terminal part (UbP_N, residues 14–141) did not. To further demonstrate which domain of DC-UbP binds with UbE1 or USP5, we performed NMR titration experiments on the ^15^N-labeled DC-UbP protein. The backbone assignment of full-length DC-UbP was just derived from the assignments of the two individual domains [13], [14]. With the stepwise addition of USP5 or UbE1 into the labeled DC-UbP, the intensities of ^1^H-^15^N cross peaks became weak or even disappearing in the spectra (Fig. S2), indicating that USP5 or UbE1 directly bound with DC-UbP. When the peak intensity (as indicated by peak height) of each amide of free DC-UbP was normalized as 1, the ratios of the peak intensities over those in DC-UbP alone at each titration step could be plotted against the residue number of DC-UbP (Fig. 2C, 2D) [16], [17]. The mean peak intensities were 0.56 and 0.27 for the UBD and UbL domains of DC-UbP, respectively, at the 1∶1 molar ratio of USP5 and DC-UbP (Fig. 2C). Similarly, the mean peak intensities for the UBD and UbL domains were 0.54 and 0.39 when titrated with UbE1 at the 1∶1 molar ratio (Fig. 2D). The C-terminal UbL domain of DC-UbP exhibited a more intensive reduction in the peak intensities than the N-terminal UBD domain, suggesting that DC-UbP bound with USP5 or UbE1 preferentially on its C-terminal UbL domain.

### The tandem UBA domains of USP5 is responsible for its binding with the UbL domain of DC-UbP

**Figure 3:**
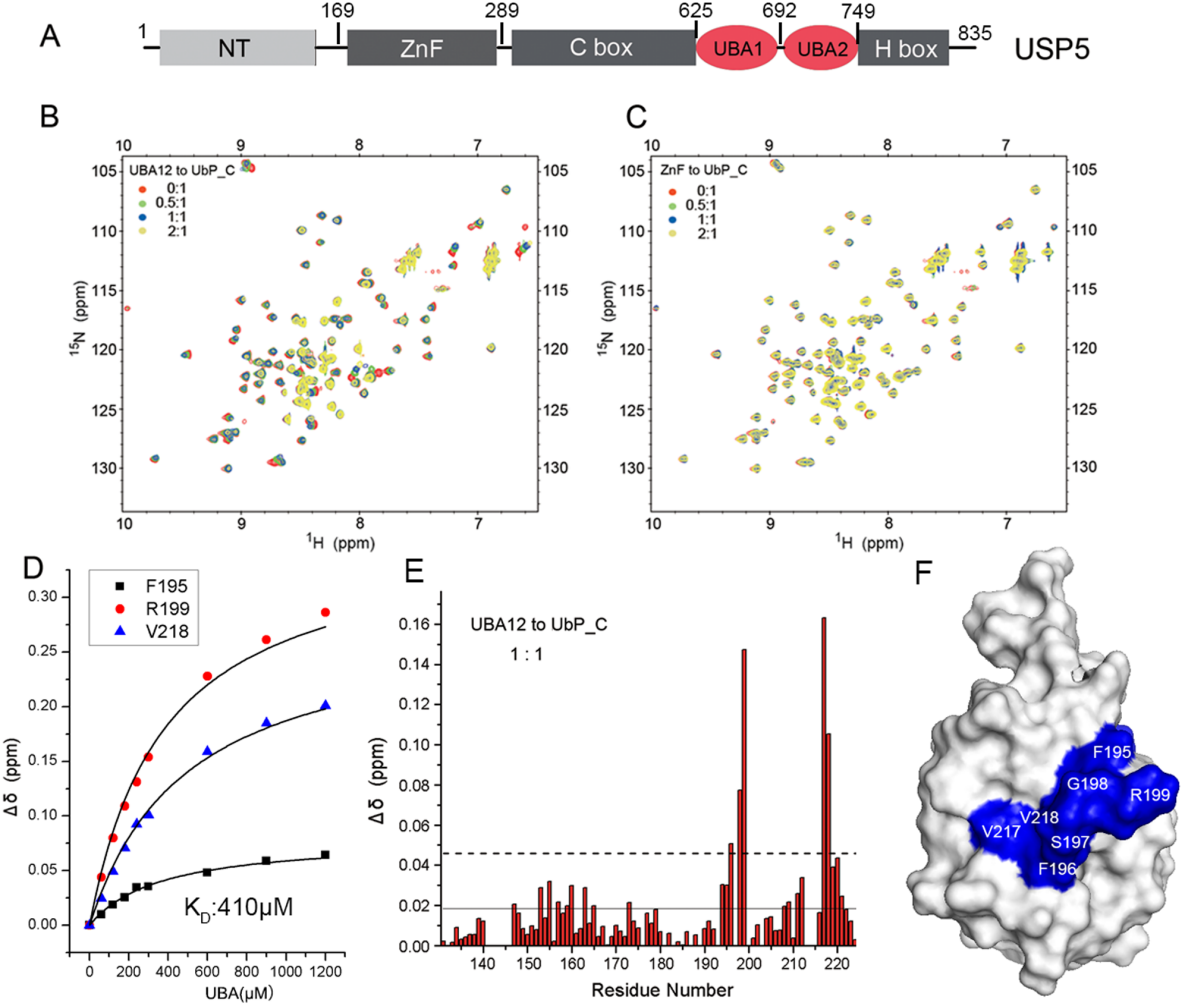
Characterization of the DC-UbP-binding domain in USP5. **A,** Domain architecture of USP5. NT, N-terminal domain; ZnF, zinc-finger domain; UBA1 and UBA2, tandem UBA domains (UBA12); C-box and H-box, two boxes of the USP domain. The constructs of USP5_ZnF, USP5_UBA12 and USP5_UBA1 include residues 169–289, 625–749 and 631–692, respectively. **B,** Overlay of the HSQC spectra of ^15^N-labeled UbP_C (200 µM) and addition of UBA12 at different molar ratios. **C,** As in (B), overlay of the HSQC spectra of ^15^N-labeled UbP_C (200 µM) and addition of ZnF at different molar ratios. **D,** Titration of UbP_C with UBA12 for measuring the binding affinity. The concentration of ^15^N-labeled UbP_C was 150 µM. **E,** Diagram of the chemical-shift changes of UbP_C upon titration with UBA12 versus residue number. The molar ratio of UBA12 over UbP_C was 1∶1. The solid and dashed lines indicate the threshold values of Mean and Mean+SD for the chemical shift changes. The residues with chemical shift changes above the Mean+SD value (dashed line) are considered involved in substantial contact with UBA12. **F,** Mapping of the UBA12-binding surface (blue) on the UbL domain. This binding-site surface is similar to that of the UBA binding on Ub molecule. The structural model was generated based on chemical-shift perturbation data and HADDOCK analysis and displayed with PyMOL.

In USP5, both of its UBA and ZnF domains bind with Ub (Fig. 3A) [19], [22]. The C-terminal portion of DC-UbP is a Ub-like (UbL) domain, but it has a distinct surface with respect to Ub [13]. So we wondered whether this UbL domain could bind the tandem UBA12 domains or the ZnF domain of USP5. We labeled UbP_C (the UbL domain of DC-UbP) with ^15^N and carried out NMR titration with USP5_UBA12 (residues 625–749) and USP5_ZnF (169–289) domains, respectively. Fig. 3B showed overlay of the ^1^H-^15^N HSQC spectra of UbP_C in the presence of different concentrations of USP5_UBA12. Some of the resonance peaks on the spectra of UbP_C shifted or disappeared with the stepwise addition of USP5_UBA12, suggesting that the UbL domain of DC-UbP interacted with the tandem UBA12 domains of USP5. However, titration with USP5_ZnF caused little chemical shift change (Fig. 3C), indicating that UbL did not interact with the ZnF domain of USP5. A rational reason is that the UbL domain of DC-UbP lacks the conserved C-terminal GG motif, which is necessary to the binding of Ub with the ZnF domain of USP5 [22]. On the other hand, binding of Ub with UBA is largely dependent on the hydrophobic surfaces [23], [24], which is present either in Ub or in UbL of DC-UbP. We also determined the binding affinity of UbP_C with tandem USP5_UBA12 by NMR titration (Fig. 3D). The average *K*_D_ value for the two UBA domains was around 400 µM. This affinity is weaker than those of Ub with USP5_UBA12 (*K*_D_ = ∼50 µM) and other UBA domains [24], [25]. Fig. 3E showed the chemical-shift perturbation of UbP_C upon titrating with USP5_UBA12 at a molar ratio of 1∶1. The residues on UbL exhibiting significant chemical-shift change or peak broadening were probably resided on the binding site. In combination of the chemical-shift perturbation data and interface analysis by HADDOCK program (Fig. S3) [26], we mapped the binding interface on the structure of the UbL domain (Fig. 3F). These residues including Phe195, Phe196, Val217 and Val218 are located at the β4 and β5 strands on the opposite surface of the positively-charged surface [13], which may contribute to the binding specificity of UbL with UBA12 of USP5.

**Figure 4:**
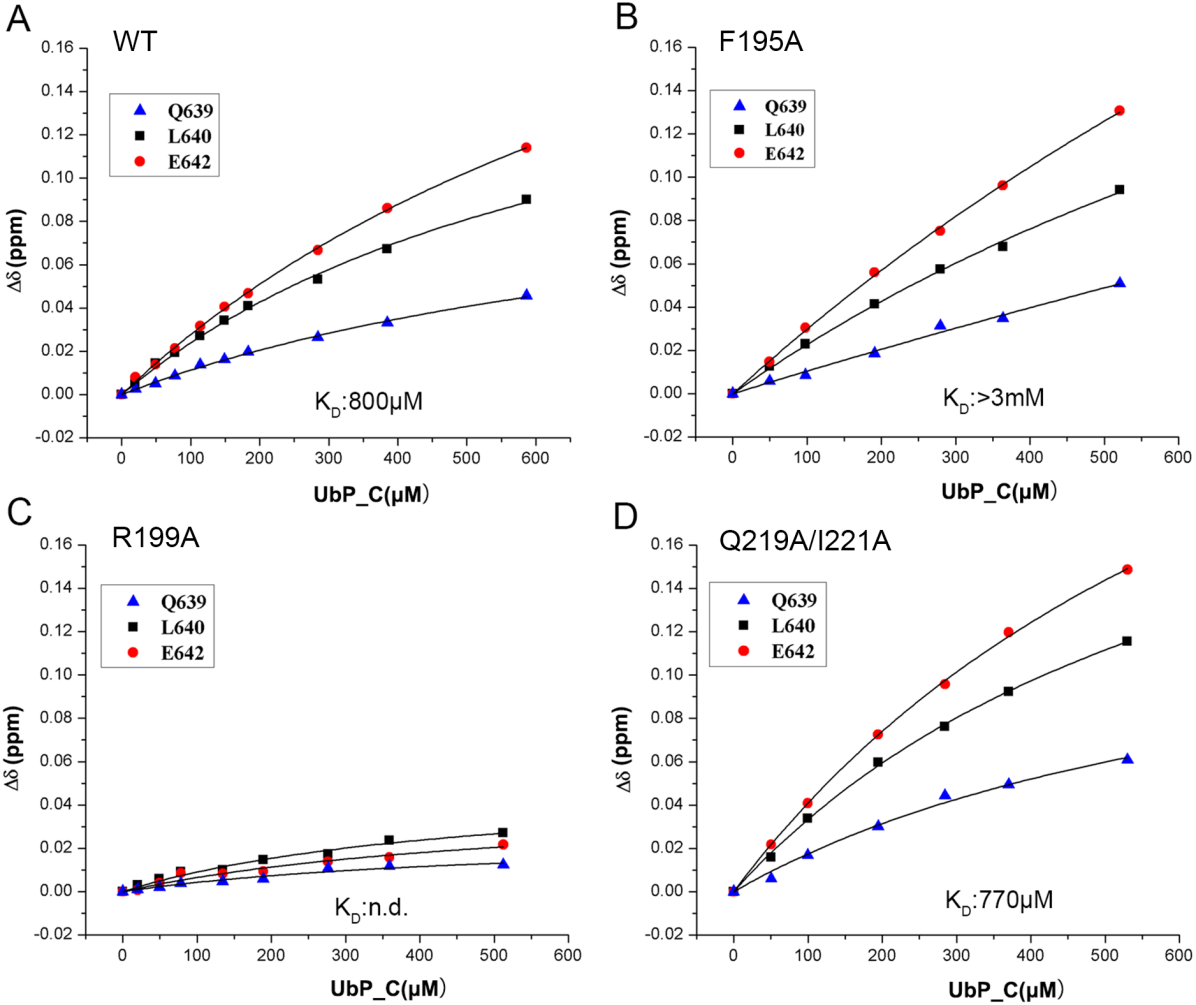
NMR titration of USP5_UBA1 with UbP_C or its mutants. **A,** Wild-type UbP_C. **B,** F195A. **C,** R199A. **D,** Q219A/I221A. The concentration of ^15^N-labeled UBA1 was 100 µM. The chemical-shift changes of residues Gln639, Leu640 and Glu642 on UBA1 were selected to plot with the increasing concentration of UbP_C. The *K*_D_ value for R199A was not obtained due to neglected chemical-shift changes. n.d., not detectable.

To substantiate the essentiality of the key residues on the UbL surface of DC-UbP for binding with USP5_UBA, we generated three mutants, F195A, R199A and Q219A/I221A; and carried out NMR titration analysis. Here we used UBA1 for NMR titration, because we thought that individual UBA and tandem UBA12 bound with UbL in a similar manner. The result showed that, compared with wild-type UbL (Fig. 4A), the F195A mutant gave a reduced binding affinity considerably with UBA1 (Fig. 4B), whereas R199A lost its binding ability (Fig. 4C). However, as a control, the Q219A/I221A mutant did not show any affinity change (Fig. 4D). It suggests that residues Phe195 and Arg199 of UbL are important to interaction with the UBA domains of USP5.

### The C-terminal UFD domain of UbE1 interacts with the UbL domain of DC-UbP

**Figure 5:**
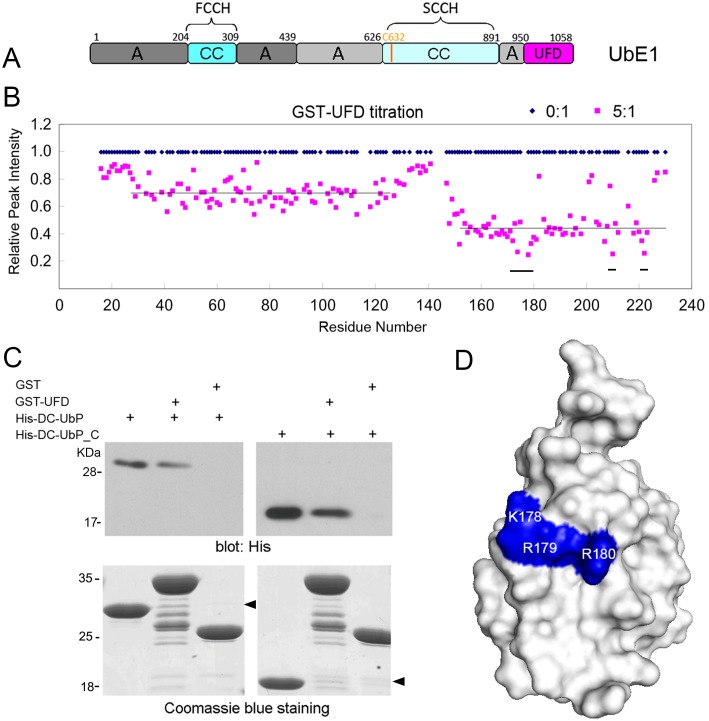
Characterization of the DC-UbP-binding domain in UbE1. **A,** Domain architecture of human UbE1. A, adenylation domain, CC, catalytic cysteine domain; FCCH, first CC half domain; SCCH, second CC half domain; UFD, Ub-fold domain. **B,** NMR titration of DC-UbP with GST-UFD. The relative peak intensities for both N- and C-terminal domains of DC-UbP were displayed upon addition of GST-UFD at the molar ratios of 0∶1 (blue) and 5∶1 (pink) into the ^15^N-labeled DC-UbP protein. The lines indicate the mean peak intensities for each domain of DC-UbP at 5∶1, which are 0.69 for the UBD domain (residues 27–126) and 0.44 for the UbL domain (152–225), respectively. The bars indicate significantly perturbed regions. **C,** GST pull-down for testing the interaction between UFD and DC-UbP or its C-terminal part. GST-UFD and His-tagged DC-UbP or UbP_C were used for the experiments, and the anti-His antibody for Western blotting. The arrows indicate the bands for pull-down proteins. **D,** Mapping of the UFD-binding surface (blue) on the UbL domain. This binding-site surface is resided on the positively-charged surface just opposite to that of the UBA binding (see Fig. 3F). The structural model was displayed with PyMOL.

Mammalian UbE1 is also a large multi-domain enzyme with unknown structure, but the 3D structures of its orthologs have been solved [27], [28]. The domain architecture of human UbE1 shows that it is mainly comprised of the adenylation domain (AD), the catalytic cysteine domains (FCCH, SCCH) and the C-terminal UFD domain (Fig. 5A) [29]. We purified three fragments of UbE1 and performed NMR titration experiments on DC-UbP and its separate domains. The individual UFD protein was not stable when purified *in* *vitro*, so we expressed and purified this domain as a GST-fused form for the experiments. The results showed that only the C-terminal UFD part bound with DC-UbP, whereas both the FH (FCCH+AD) and SCCH fragments did not (Fig. S4A–S4C). Similar with USP5 (Fig. 3), the UFD domain of UbE1 directly interacted with DC-UbP, preferentially on the UbL domain of DC-UbP but not on the N-terminal UBD domain (Fig. 5B). Further NMR titration experiments with individual parts of DC-UbP verified that only the C-terminal UbL domain bound with UFD (Fig. S4D, S4E). GST pull-down experiment confirmed that the UFD domain of UbE1 directly interacted with DC-UbP (Fig. 5C, lane 2) and especially its C-terminal UbL domain (Fig. 5C, lane 5). We also attempted to map the interacting surface on the UbL structure from NMR titration data (Fig. 5B). The residues with significant intensity decreases include Val174, K178 and R179, and Leu210 and Val222. It is known that the UFD domain of yeast Uba1 contains an acidic surface that specially interacts with the positive charges on the surface of UbE2 (Fig. S5) [27]. Thus, we presumed that the UFD-domain binding site on the UbL domain is mainly located on the positively-charged surface including residues Lys178, Arg179 and Arg180 (Fig. 5D) [13], which is opposite to the UBA binding site on UbL (Fig. 3F). This provides a possibility that USP5 and UbE1 bind to the UbL domain of DC-UbP simultaneously but on the opposite position.

### UbE1 and USP5 still reserve their respective catalytic activities when bound with DC-UbP

**Figure 6:**
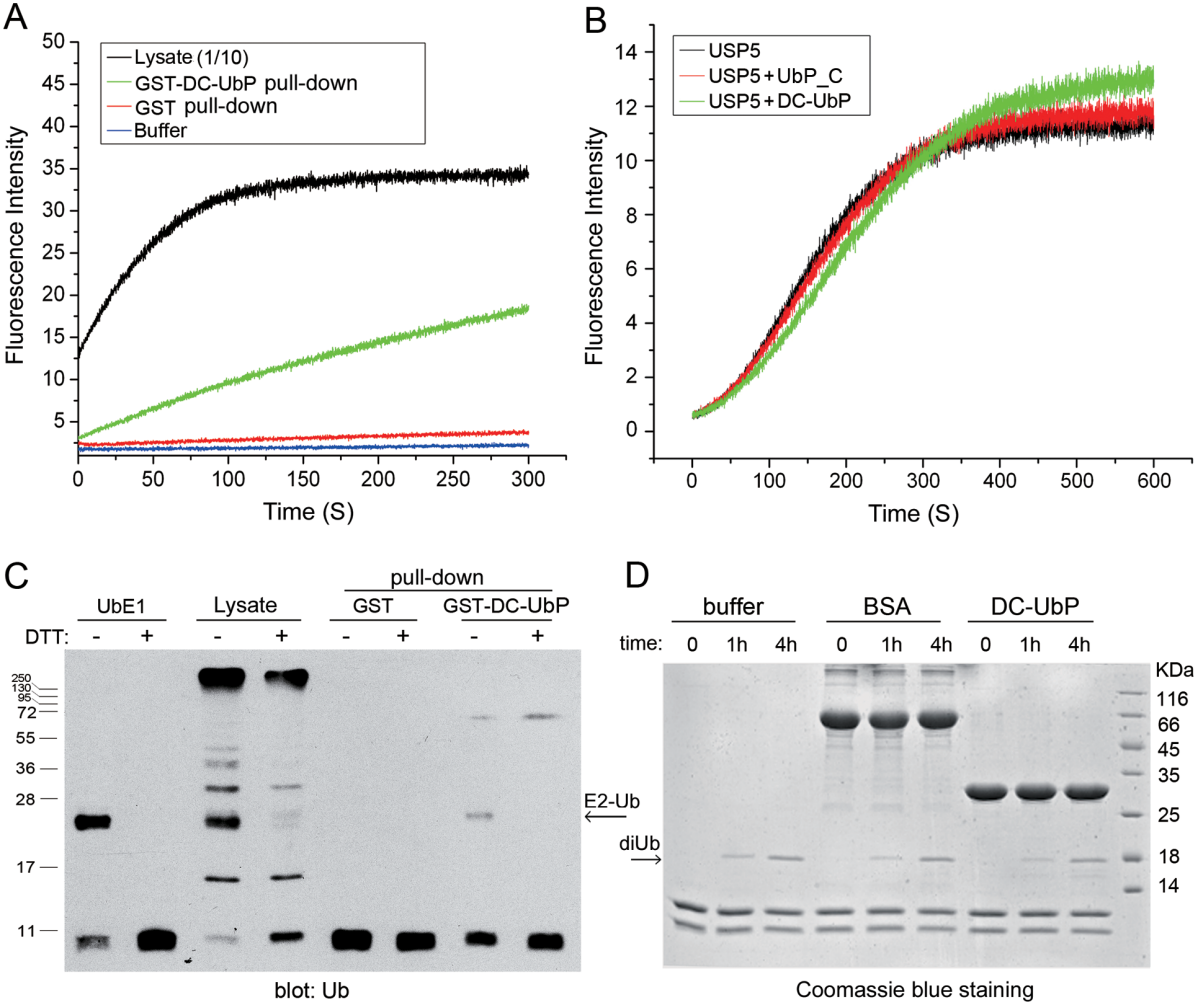
Effects of DC-UbP binding on the catalytic activities of UbE1 and USP5. **A,** Fluorescence assay for deubiquitinating activity of the components from GST-DC-UbP pull-down. The GSH conjugated beads were incubated with purified GST-DC-UbP and HEK 293T cell lysates and then eluted with GSH. The components that may contain the USP5 enzyme were subjected to activity assay with Ub-AMC (250 nM) as a substrate. The cell lysates was set as a positive control; and the GST pull-down components was a negative control. **B,** Fluorescence assay for deubiquitinating activities of purified USP5 in the presence of DC-UbP or UbP_C. USP5 (10 nM) was pre-incubated with 10 mM DTT for 15 min, then DC-UbP or UbP_C was added with 10-fold excess in amounts. **C,** *In* *vitro* ubiquitin conjugation assay for the components from GST-DC-UbP pull-down. The pull-down components were subjected to assaying the activity of UbE1 enzyme (lanes 7 and 8). Purified UbE1 and cell lysates were set as positive controls, and the GST pull-down components were a negative control. In this reaction, the UbcH5C-Ub (E2-Ub) conjugates were generated, while dissociated in the presence of DTT. **D,** Effect of purified DC-UbP on the diUb synthesis *in* *vitro*. The concentrations of UbE1 and E2-25K were 0.25 µM and 2 µM, respectively, while those of His_6_-DC-UbP and BSA were 80 µM. The reaction system (100 µL) included 0.25 mg Ub, 0.25 mg Ub-K48R and 2 mM ATP in a buffer of 50 mM Tris, 10 mM MgCl_2_, pH 8.0.

UbE1 and USP5 are two enzymes functioning in the ubiquitination and deubiquitination processes, respectively. Their catalytic activities can be monitored by several established methods *in* *vitro* [18], [20]. We firstly examined whether the components pulled down by GST-DC-UbP from cell lysates still reserved the activity of catalyzing hydrolysis of Ub-AMC. The untreated cell lysates (with 10-fold dilution) were set as a positive control, while the GST pull-down sample was as a negative control. The increase of fluorescence intensity represents the hydrolysis of Ub-AMC. Fig. 6A displayed the time courses of fluorescence intensity changes upon addition of different pull-down samples. The data clearly demonstrated that the components pulled down by GST-DC-UbP (from cell lysates) could hydrolyze Ub-AMC, albeit the activity seemed to be lower than that of the untreated cell lysates. This indicates that the native USP5 enzyme pulled down by GST-DC-UbP from the cell lysates still reserves its catalytic activity on Ub-AMC. It also implies that binding with DC-UbP may have little impact on the USP5 activity. To verify this speculation, we purified the USP5 protein and monitored the effect of DC-UbP or its UbL domain on the enzymatic activity. The kinetic profiles showed that the deubiquitinating activity of USP5 was not affected significantly even in the presence of more than ten-fold excess of DC-UbP or UbP_C (Fig. 6B and Fig. S6). Therefore, DC-UbP binding has no detectable effect on the catalytic activity of USP5.

We also measured the Ub activating activities of the components pulled down by GST-DC-UbP from cell lysates by detecting the Ub conjugation to UbcH5C (UbE2). As a positive control, the purified UbE1 could activate Ub and conjugate Ub to UbcH5C to form a thioester linkage (Fig. 6C, lane 1). The activating activity could also be detected from the untreated cell lysates (with 10-fold dilution) (Fig. 6C, lane 3), suggesting that the endogenous UbE1 had its activity of catalyzing activation of Ub for transferring to UbE2. Besides UbE2 thioester, the DTT-resistant species, like diUb, tetraUb or other higher molecular-weight Ub chains could also be observed due to potential contaminant of E3 ligase from the cell lysates. For the components pulled down by GST-DC-UbP, this sample still had the activities of activation and conjugation of Ub to UbE2 (Fig. 6C, lane 7), whereas, under the same condition, the components from GST pull-down did not possess this action. These data further support the specific interaction between DC-UbP and UbE1, and more importantly, UbE1 reserves the activating activity in its DC-UbP-associated form. We then carried out the experiment on synthesis of diUb chain *in* *vitro* to examine whether the presence of excess DC-UbP affects the downstream of ubiquitination by UbE1 activity (Fig. 6D). By detecting formation of K48-linked diUb at different incubation time, it was obvious that DC-UbP had no detectable effect on the reaction process. Taken together, all the data demonstrate that UbE1 reserves its activating activity when it is bound with DC-UbP.

### Overexpression of DC-UbP improves the association between UbE1 and USP5 in cell

**Figure 7:**
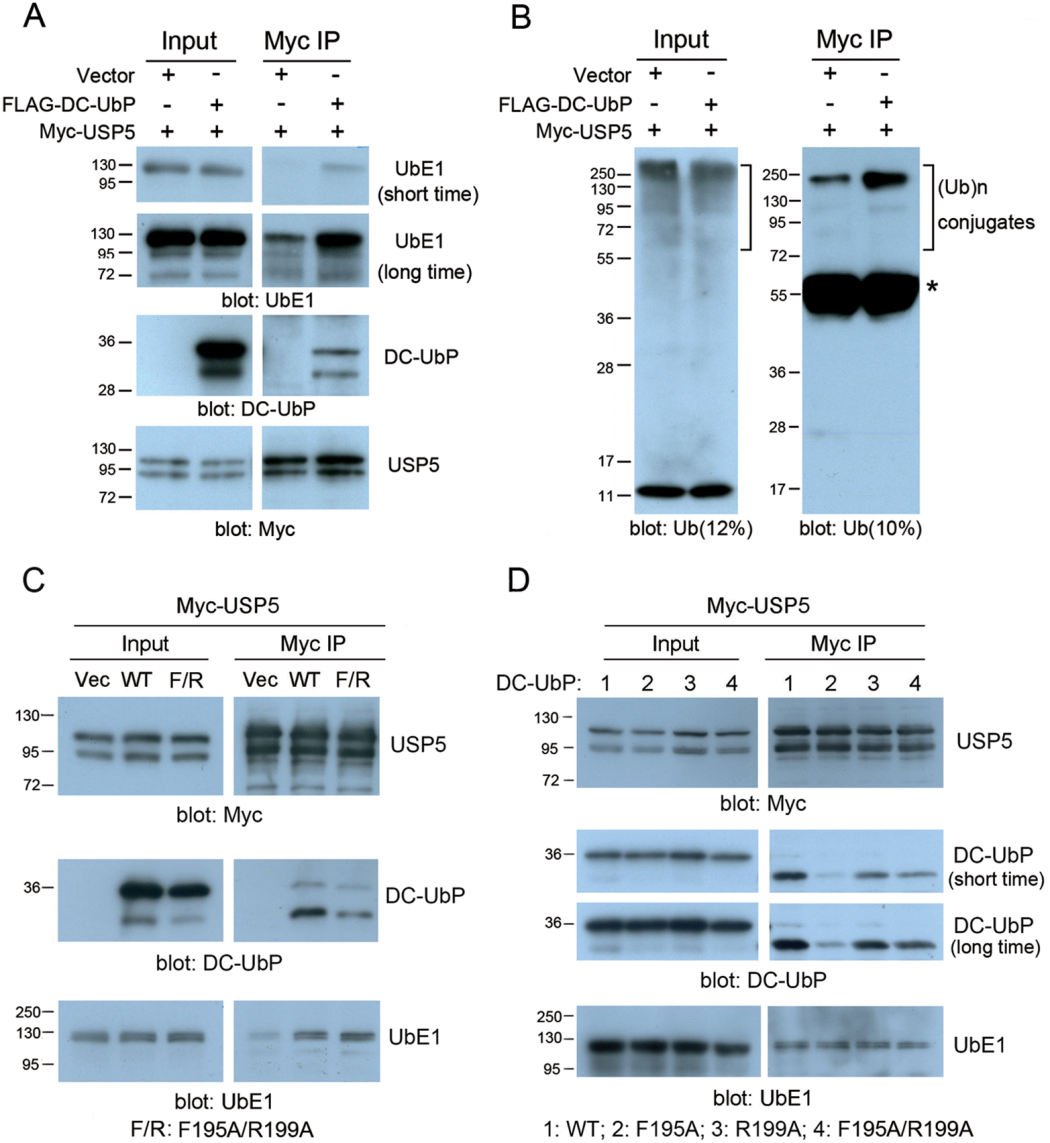
Effects of DC-UbP overexpression on the association of UbE1 and USP5. **A,** Co-IP for detecting the association of UbE1, USP5 and DC-UbP. Myc-USP5 was co-transfected with FLAG-DC-UbP or mock vector into HEK 293T cells and then precipitated with anti-Myc antibody. The endogenous UbE1 was monitored by anti-UbE1 antibody. **B,** As in (**A**), detected with Ub antibody. After IP, the Ub conjugate level and especially the 250-kDa species were increased when co-transfected with DC-UbP. *, IgG heavy chain. **C,** Effect of the F195A/R199A mutant of DC-UbP on the association of USP5 and UbE1. Due to partial removal of the FLAG tag during co-IP experiment, two bands for DC-UbP by an antibody against DC-UbP were observed. **D,** As in (**C**), effect of the DC-UbP mutants on the association of USP5 and UbE1. Myc-USP5 was co-transfected with FLAG-tagged WT or its mutants (F195A, R199A, and F195A/R199A) and analyzed by Western blotting. The gels were blotted with anti-Myc, anti-DC-UbP and anti-UbE1 antibodies, respectively. Due to proteolytic degradation, two bands were detected in the gel by anti-DC-UbP antibody.

Because association of DC-UbP did not affect the catalytic activities of UbE1 and USP5 *in* *vitro*, we wondered whether DC-UbP association had any effects on the cellular functions of these two enzymes. We previously observed that FLAG-tagged DC-UbP could immunoprecipitate both UbE1 and USP5 in HEK 293T cells (Fig. 2B). So we asked whether USP5 immunoprecipitate endogenous UbE1 under the condition of DC-UbP overexpression. When co-transfected with FLAG-DC-UbP, Myc-USP5 immunoprecipitated more UbE1 than that with the mock (Fig. 7A). We then detected total Ub conjugates of the cells transfected with FLAG-DC-UbP. The Ub conjugation level in the cell lysates was not changed obviously for co-transfection of Myc-USP5 with FLAG-DC-UbP. However, after immunoprecipitation with the anti-Myc antibody, the Ub conjugation level was considerably increased as compared to the vector control (Fig. 7B), especially the conjugates with large molecular weights in the gel, suggesting formation of a functional complex mediated by DC-UbP. Because DC-UbP binds with USP5 and UbE1 directly, the increased amount of DC-UbP provides a mediator for association between UbE1 and USP5, in which some ubiquitinated conjugates are included. We then produced three mutants on the UbL surface of DC-UbP (F195A, R199A and F195A/R199A) that could destroy the interaction with USP5. Indeed, these mutations somewhat reduced the interactions with USP5 (Fig. 7C, 7D, middle panels); however, they had no significant effect on the association of USP5 with endogenous UbE1 (Fig. 7C, 7D, bottom). This is probably that endogenous DC-UbP or other factors in cell are also involved in mediating the association between USP5 and UbE1. All these data suggest that DC-UbP mediates association between USP5 and UbE1 in cell through directly interacting with these two enzymes.

### Overexpression of DC-UbP modulates the cellular ubiquitination level

**Figure 8:**
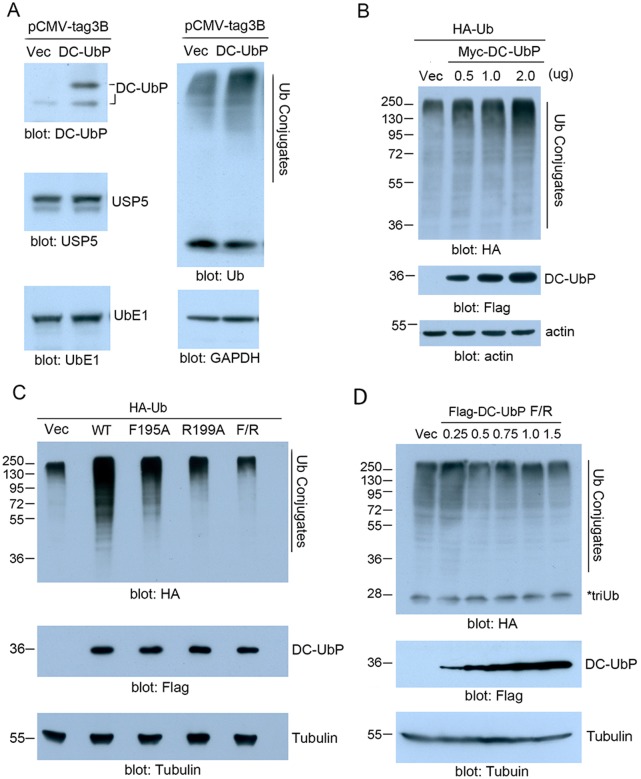
Overexpression of DC-UbP alters the total level of Ub conjugates in HEK 293T cells. **A,** Effect of DC-UbP overexpression on the total level of Ub conjugates. The transfected plasmid for DC-UbP was 0.8 µg for one well of a 6-well plate. The USP5 and UbE1 levels remained unchanged. GAPDH expression worked as a reference. **B,** Dose-dependent assay for the level of the Ub conjugates with the increase of DC-UbP plasmid (0, 0.5, 1.0 and 2.0 µg DNA). HA-Ub was co-transfected with DC-UbP and blotted with anti-HA antibody. **C,** DC-UbP mutation caused a reduced effect on the level of Ub conjugates. The amount of plasmid for DC-UbP or its mutants was ∼0.6 µg. F/R, F195A/R199A. **D,** Dose-dependent assay for the Ub conjugation level with the increase of F195A/R199A.

To get information whether DC-UbP impacts on the cellular ubiquitination level, we overexpressed DC-UbP and detected the Ub conjugates in HEK 293T cells. When the cells were transfected with the plasmid for DC-UbP at an appropriate amount, the total level of the Ub conjugates increased considerably as compared with the vector control (Fig. 8A), whereas the levels of its interacting partners, USP5 and UbE1, remained unchanged. We then performed a dose-dependent assay, and found that the level of Ub conjugates increased gradually with the increase of DC-UbP transfection (Fig. 8B). However, the R199A and F195A/R199A mutants of DC-UbP lost this effect to increase the conjugation level (Fig. 8C). Further extensive study by dose-dependent experiment showed that the double mutant totally lost this modulating effect on the Ub conjugation level (Fig. 8D). Taken together, these results demonstrate that DC-UbP modulates the cellular ubiquitination levels depending on its cellular amount, which is probably through mediating the association between USP5 and UbE1.

### Knockdown of DC-UbP reduces the cellular ubiquitination level

**Figure 9:**
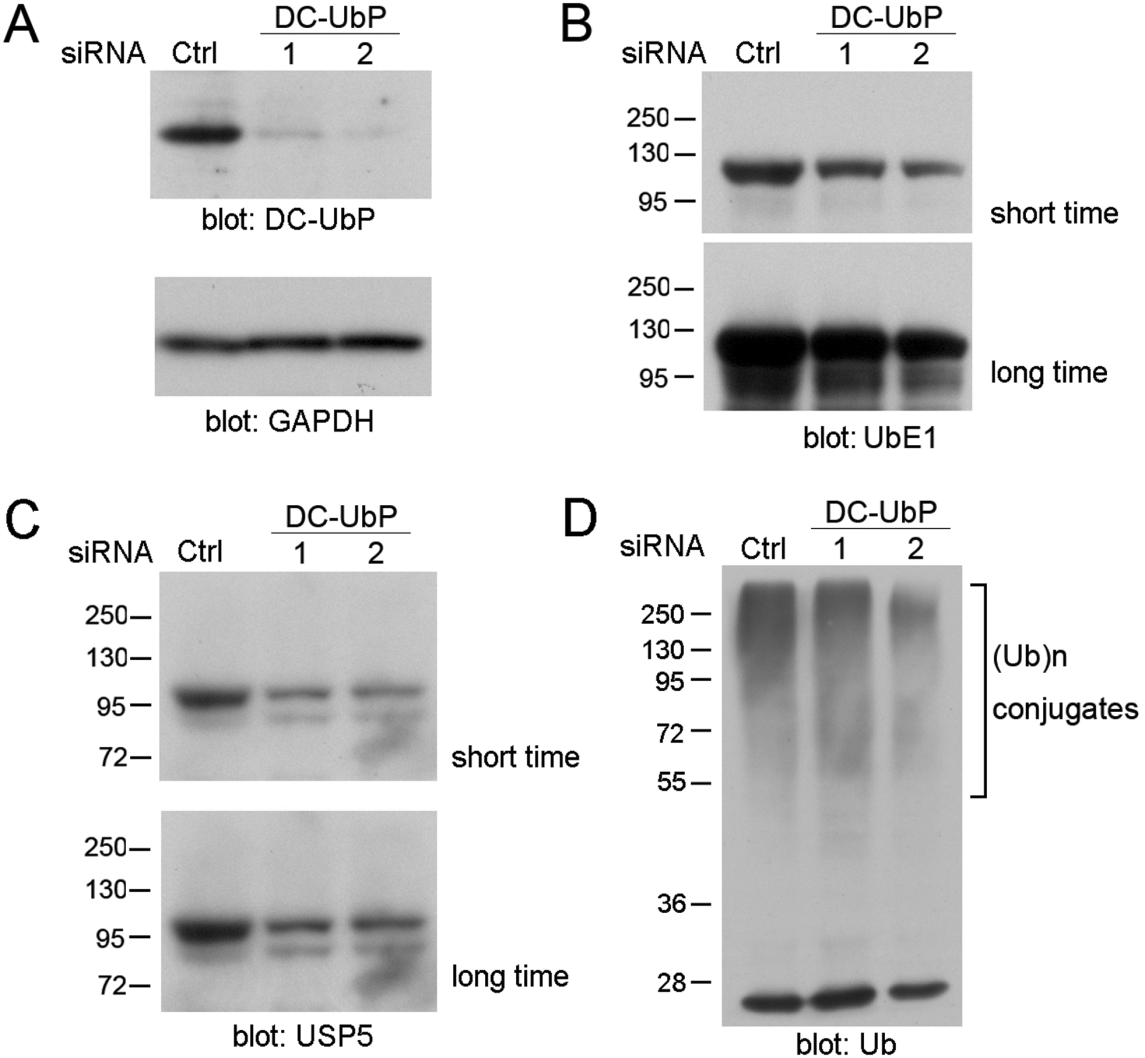
Effect of DC-UbP knockdown on the cellular ubiquitination level. **A,** Knockdown of DC-UbP by siRNAs in HEK 293T cells. **B,** Detection of UbE1 after knockdown of DC-UbP. The Western blotting for the cell lysates was performed with an anti-UbE1 antibody. **C,** As in (**B**), detection of USP5 after knockdown of DC-UbP with an anti-USP5 antibody. **D,** As in (**B**), detection of the Ub conjugates after knockdown of DC-UbP with an anti-Ub antibody. The Ub conjugate level was reduced when DC-UbP was silent. GAPDH was applied for a loading control.

When knockdown of DC-UbP in HEK 293T cells by siRNAs (Fig. 9A), the amount of UbE1 was decreased considerably as detected with the antibody against UbE1 (Fig. 9B). Similarly the USP5 level was also reduced as the anti-USP5 antibody indicated (Fig. 9C). This indicates that formation of the DC-UbP complex may be beneficial to the stabilities of both UbE1 and USP5. Intriguingly, the ubiquitinated conjugate level was also reduced in the cell lysates (Fig. 9D). It is possible that reduction of the UbE1 enzyme may result in decrease of the ubiquitination level and consequently the ubiquitinated conjugates in cells [30]. Although the amount of USP5 is simultaneously decreased when knockdown of DC-UbP in cell, we are still not clear whether this enzyme is contributable to regulating the ubiquitination level. Since USP5 is known to function mainly in hydrolyzing unanchored Ub chains or Ub precursors [9], it is reasonable to presume that reduction of USP5 may affect the Ub pool but not the ubiquitinated substrates.

## Discussion

### Cross-talk between protein ubiquitination and deubiquitination

Protein ubiquitination and deubiquitination are two reversible processes that control the cellular homeostasis of various Ub forms, including free Ub, Ub precursors, unanchored Ub chains and ubiquitinated substrates [9]. Accumulating evidence shows that some ubiquitination enzymes can interplay with deubiquitinating enzymes and mediate the cross-talk between these two opposite processes. The DUB ataxin-3 coordinates with the E2 enzyme Ube2w in regulating the ubiquitination cycle of CHIP [31]; while another DUB, OTUB1, interacts with the E2 enzyme UBC13 to inhibit the RNF168 pathway for DNA damage response [32], [33]. Some DUB enzymes can also bind with an E3 ligase, such as USP8 with Nrdp1 [34] and USP7 with ICP0 [35], and regulate the stability and activity of the E3 ligase. More interestingly, the deubiquitinating enzyme A20 contains both Ub ligase and DUB domains that cooperatively down-regulates NF-κB signaling [36]. Our finding that DC-UbP/UBTD2 couples UbE1 and USP5 together to form a functional complex will exemplify the cross-talk between ubiquitination and deubiquitination processes that orchestrates the ubiquitin pool in cell.

### Both UbE1 and USP5 are involved in the homeostasis of ubiquitin pool

UbE1 (Uba1) is central to Ub conjugation since it initiates the first key step of ubiquitination process by catalyzing Ub activation [1]. UbE1 is relatively unique for Ub conjugation and highly conserved in all eukaryotes [37]. USP5 is an important DUB enzyme that mainly disassembles the unanchored polyUb chains or Ub precursors to regenerate free Ub molecules [38], [39]. As its yeast homologue Ubp14 [40], USP5 can sequentially remove Ub from the proximal end of unanchored polyUb chains [41]. Thus, it is reasonable to deduce that both UbE1 and USP5 are contributable to maintaining the cellular homeostasis of Ub pool.

Both USP5 and UbE1 are large proteins with multi-domains involved in regulating cellular ubiquitination levels. Structurally, USP5 is constituted by two ZnF-UBP domains [42], a catalytic USP domain and two inserted UBA domains [22]. All the four Ub-binding domains participate in binding with polyUb chain in the cleavage process [22], [43]. Our study suggests that DC-UbP binds to USP5 on the tandem UBA domains, implying that binding of DC-UbP to USP5 potentially regulates the deubiquitination process. On the other hand, the UbE1 enzyme utilizes ATP to activate the terminal glycine residue of Ub generating a covalent thioester linkage between ‘activated’ Ub and the UbE1 enzyme itself [44]. The UbE1 protein is mainly comprised of three domains, the adenylation domain that binds ATP and Ub, the catalytic cysteine domain that binds activated Ub, and the C-terminal Ub-fold domain (UFD) that recruits specific E2 conjugating enzymes [27]. Our finding that DC-UbP binds to UbE1 on the C-terminal UFD domain suggests that DC-UbP also participates in regulating the ubiquitination process. Thus, coordination of UbE1 and USP5 by DC-UbP is involved in modulating the Ub pool in cell.

### DC-UbP links UbE1 and USP5 enzymes to modulate the ubiquitin pool

**Figure 10:**
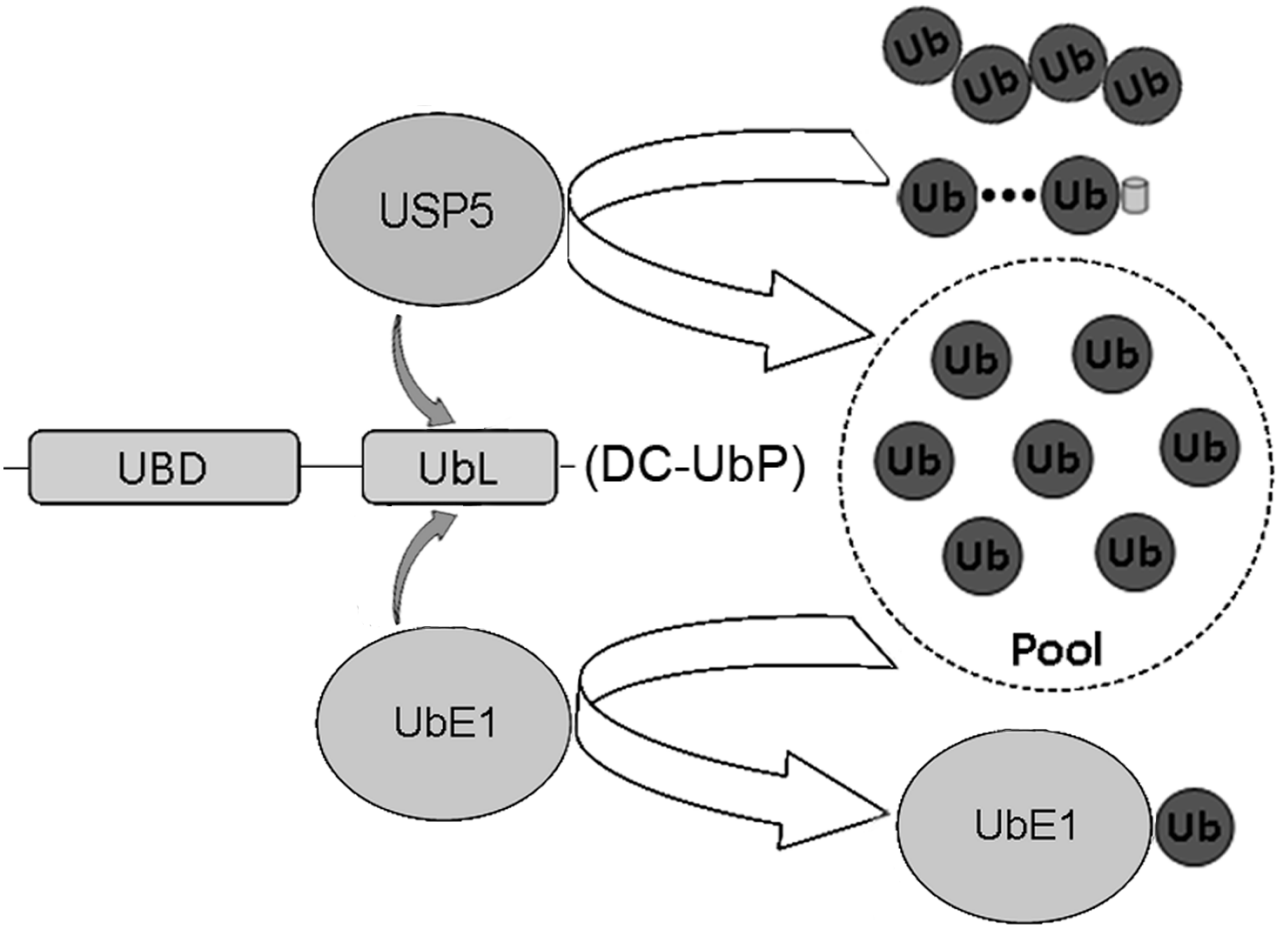
Schematic representation for DC-UbP coupled ubiquitination and deubiquitination in cell. In this cartoon, DC-UbP mediates USP5 and UbE1 to form a population of complex through transient interactions. This dynamic complex can integrate the functions of USP5 and UbE1. USP5 processes the unanchored polyUb chains or Ub precursors to constitute the free Ub pool, while UbE1 activates the Ub molecules from the pool for further conjugating to the substrates. Thus, DC-UbP reconciles the two opposite processes, ubiquitination and deubiquitination, through linking the UbE1 and USP5 enzymes.

Ub is a small protein modifier for huge number of proteins involved in myriad biological processes, such as DNA repair, gene regulation, protein degradation, translocation, apoptosis, and immune response [1], [2], [5], [45]. Maintaining the balance of the free Ub pool is critical to the eukaryotic cells [46], [47]. USP5 is a common deubiquitinating enzyme mainly responsible for the cleavage of unanchored polyUb chains or the Ub precursor proteins, and thus plays a role in maintaining the homeostasis of free Ub pool. The free Ub molecules stored in the pool can then be utilized in the next cycle and activated by UbE1 to participate in the consequent processes, conjugation to UbE2 and transfer to specific substrate by E3 ligase. We revealed that DC-UbP directly interacts with USP5 and UbE1 to form a dynamic complex through transient interactions, which can improve their stabilities, mediate their associations and integrate their functions. Our finding suggests that DC-UbP is involved in modulating the Ub pool in cells, although the underlying molecular mechanism is not clearly understood. Thus, DC-UbP modulates the cellular levels of ubiquitinated conjugates by coordinating USP5 and UbE1. These two reverse processes, ubiquitination and deubiquitination, are coupled together and interplay with each other by assistance of DC-UbP (Fig. 10). In this model, UbE1 and USP5 can bind to the C-terminal UbL domain of DC-UbP but on the opposite surface (Fig. 3F and Fig. 5D), forming a population of complex. This dynamic complex organized by DC-UbP can cooperatively integrate the functions of UbE1 and USP5, which may improve the efficiency of cellular deubiquitination and ubiquitination processes.

In conclusion, DC-UbP mediates USP5 and UbE1 to form a functionally dynamic complex that plays a regulatory role in facilitating the efficiency of ubiquitination and deubiquitination in cellular metabolism. As aberrant regulation of USP5 and UbE1 has been implicated in some diseases [30], [48], the USP5 and UbE1 enzymes have been highlighted as new therapeutic targets, and several small molecule inhibitors have been developed [49], [50]. Because DC-UbP can integrate the functions of these two enzymes, targeting DC-UbP by inhibitors might become a promising therapeutic strategy for the relevant diseases.

## Supporting Information

Figure S1 **LC-MS/MS analysis of different protein bands in SDS-PAGE gels from the GST-DC-UbP pull-down components. A**, A representative profile for separating the peptides digested from a 250-kDa protein band. The RP-C18 column (0.15 mm×150 mm, Column Technology Inc.) and the Zorbax 300 SB-C18 peptide traps (Agilent Technologies) were used for separation and collection of the digested peptides. **B**, A representative profile of the mass spectrometry for one of the peptide fragments. The MS data were further processed using BIOWORKS software to give a peak list files and the protein database of HUMAN (Version3.36) were selected for searching. **C**, Top four proteins identified from MS analysis of the two bands around 250 kDa, which include UBTD2, USP5, UbE1 and UBB (or UBC, RPS27A).
(TIF)

Figure S2 **NMR titration for charactering the interactions of DC-UbP with USP5 and UbE1. A**, Chemical-shift assignment of the DC-UbP protein. The ^1^H-^15^N HSQC spectrum shows the resonance peaks of almost all amides of full-length DC-UbP. The assignment is derived from the chemical-shift assignments of the individual UbP_N (PDB: 2KSN) and UbP_C (PDB: 1TTN) fragments that have been completed. **B**, Overlay of the HSQC spectra of ^15^N-labeled DC-UbP (100 µM) and addition of USP5 at different molar ratios. The peak broadening during USP5 titration indicates direct interaction between DC-UbP and USP5. **C**, As in (**B**), UbE1 titration. The peak broadening during UbE1 titration indicates direct interaction between DC-UbP and UbE1.
(TIF)

Figure S3 **Ribbon representation of the structure of UbL-UBA1 complex as constructed by using HADDOCK method.** The interfaces between UbL and UBA1 are shown. As known, for UBA1, the ^666^MGV^668^ loop between helix1 and helix2 contributes to the specific interaction. The residues Arg199, Gln219 and Ile221 are potentially located in the interface for the UbL domain of DC-UbP. Combined with mutagenesis, we thus characterized the residues Phe195 and Arg199 of UbL that are important to interacting with UBA1.
(TIF)

Figure S4 **NMR titration for charactering the interactions of DC-UbP with various fragments of UbE1. A**, Overlay of the HSQC spectra of ^15^N-labeled DC-UbP (100 µM) and addition of FH (FCCH and AD) fragment at different molar ratios. There is no considerable peak change in the spectra during titration with the FH fragment. **B**, As in (**A**), SCCH titration. There is no considerable peak change in the spectra during titration with the SCCH fragment. **C**, As in (**A**), GST-UFD titration, except that the concentration of ^15^N-labeled DC-UbP was 20 µM. With addition of GST-UFD, some peaks in the C-terminal part (UbL) of DC-UbP become weak or disappearing, indicating that UFD specifically binds to the UbL domain. **D**, Diagram of the peak intensity (height) changes of UbP_N titrated with GST-UFD at a molar ratio of 1∶3 against residue number. **E**, As in (**D**), diagram of UbP_C titrated with GST-UFD at a molar ratio of 1∶3. The lines indicate the mean peak intensities for the UBD (a.a. 27–126) and UbL (152–225) domains, respectively, suggesting that UFD specifically binds with UbL but not with UBD.
(TIF)

Figure S5 **Structural model of the UFD domain of human UbE1 showing the electrostatic surface.** The acidic residues on the surface corresponding to those in yeast Uba1 potentially binding to the positively-charged interface of Ubc1 (UbE2) are highlighted. The structure was generated by homology modeling using I-TASSER server and the electrostatic surface was displayed with MOLMOL.
(TIF)

Figure S6 **Effect of the C-terminal part of DC-UbP (UbP_C) on the deubiquitinating activity of USP5.** The fluorescence increases were monitored for the deubiquitinating activities of purified USP5 in the presence of different molar ratios of UbP_C/USP5. The concentrations of USP5 and Ub-AMC were 10 nM and 250 nM, respectively.
(TIF)
