# Supplementary figures and images for: A Ubiquitin Shuttle DC-UbP/UBTD2 Reconciles Protein Ubiquitination and Deubiquitination via Linking UbE1 and USP5 Enzymes
Source: PLoS One. 2014 Sep 10;9(9):e107509. doi: 10.1371/journal.pone.0107509 (PMC4160250; doi:10.1371/journal.pone.0107509)

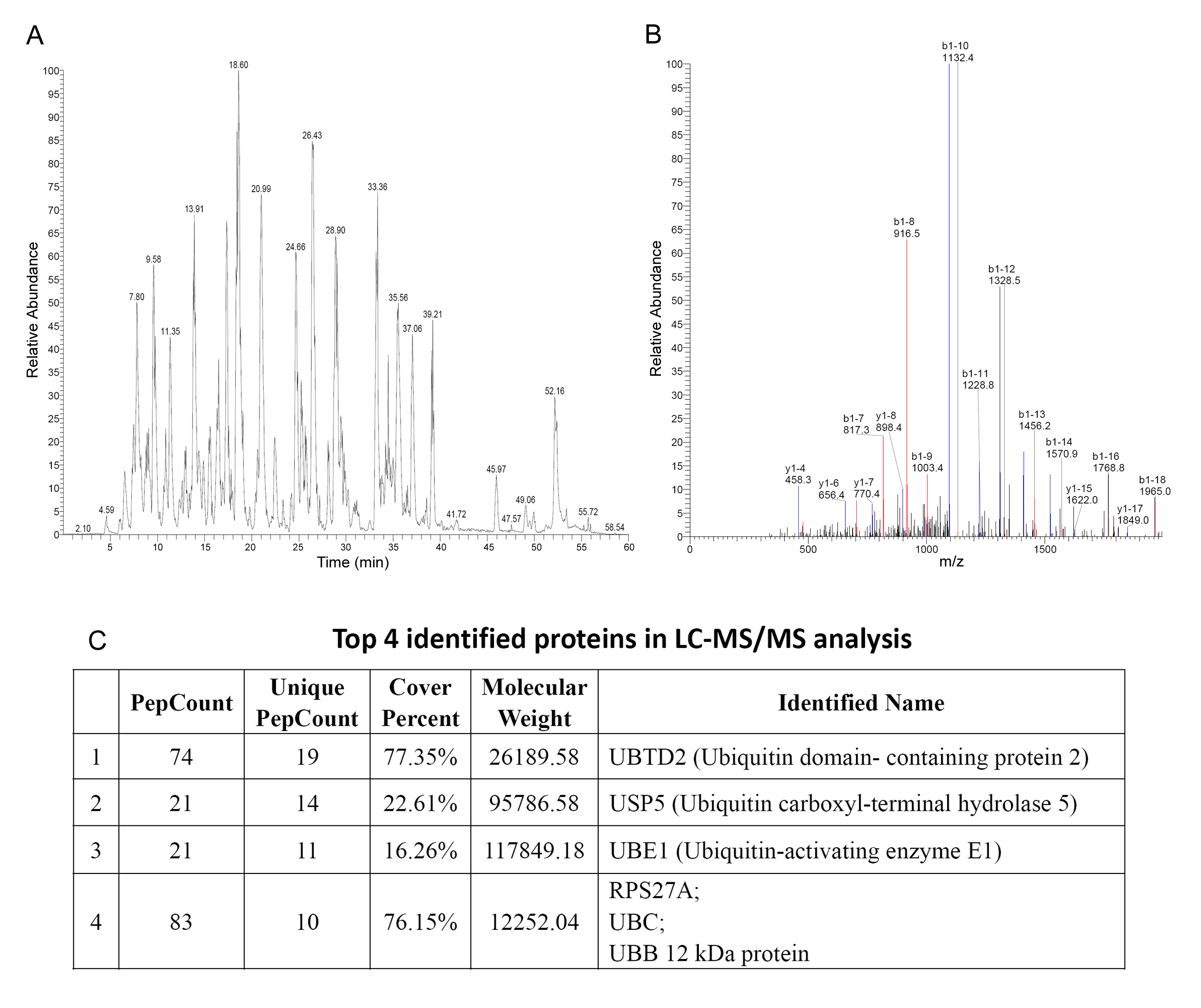

Supplement: Figure S1 — LC-MS/MS analysis of different protein bands in SDS-PAGE gels from the GST-DC-UbP pull-down components. A, A representative profile for separating the peptides digested from a 250-kDa protein band. The RP-C18 column (0.15 mm×150 mm, Column Technology Inc.) and the Zorbax 300 SB-C18 peptide traps (Agilent Technologies) were used for separation and collection of the digested peptides. B, A representative profile of the mass spectrometry for one of the peptide fragments. The MS data were further processed using BIOWORKS software to give a peak list files and the protein database of HUMAN (Version3.36) were selected for searching. C, Top four proteins identified from MS analysis of the two bands around 250 kDa, which include UBTD2, USP5, UbE1 and UBB (or UBC, RPS27A). (TIF) [file pone.0107509.s001.tif]

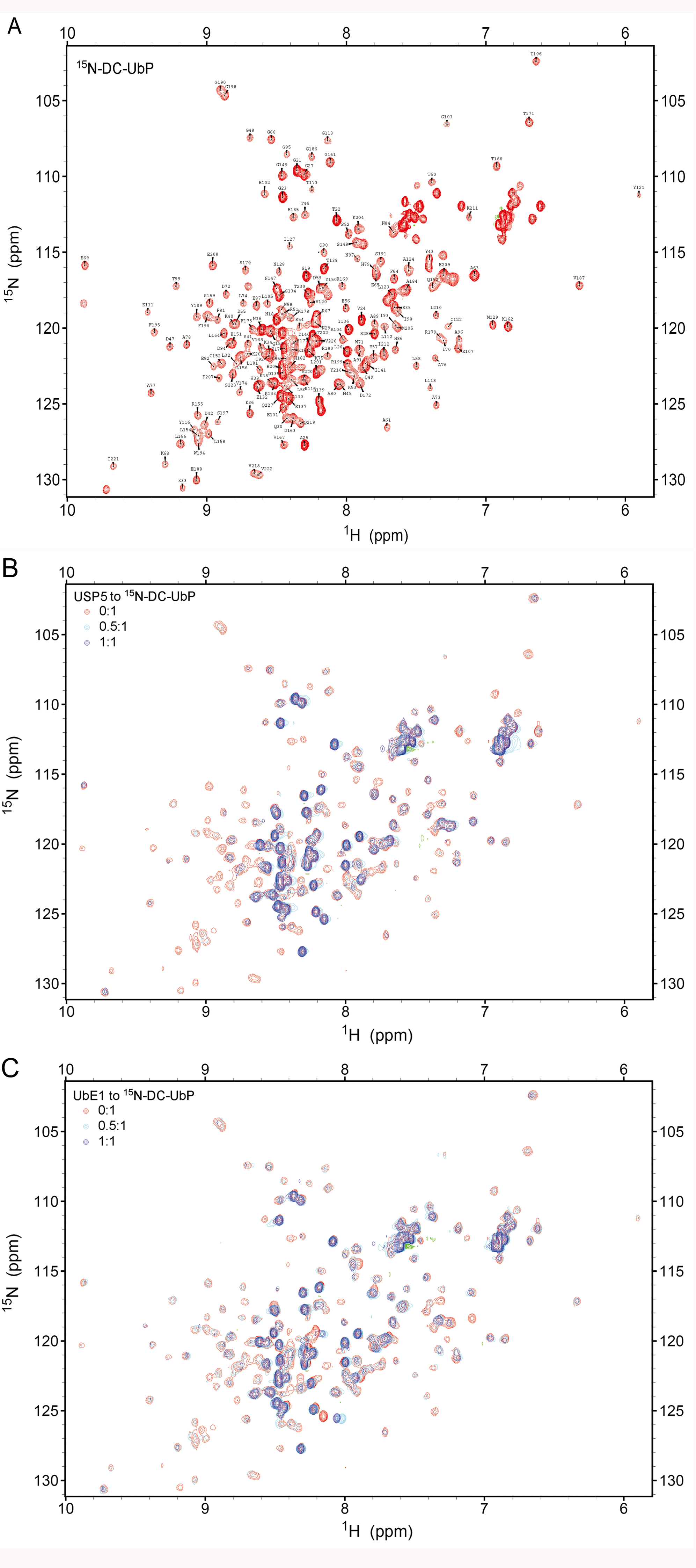

Supplement: Figure S2 — NMR titration for charactering the interactions of DC-UbP with USP5 and UbE1. A, Chemical-shift assignment of the DC-UbP protein. The 1H-15N HSQC spectrum shows the resonance peaks of almost all amides of full-length DC-UbP. The assignment is derived from the chemical-shift assignments of the individual UbP_N (PDB: 2KSN) and UbP_C (PDB: 1TTN) fragments that have been completed. B, Overlay of the HSQC spectra of 15N-labeled DC-UbP (100 µM) and addition of USP5 at different molar ratios. The peak broadening during USP5 titration indicates direct interaction between DC-UbP and USP5. C, As in (B), UbE1 titration. The peak broadening during UbE1 titration indicates direct interaction between DC-UbP and UbE1. (TIF) [file pone.0107509.s002.tif]

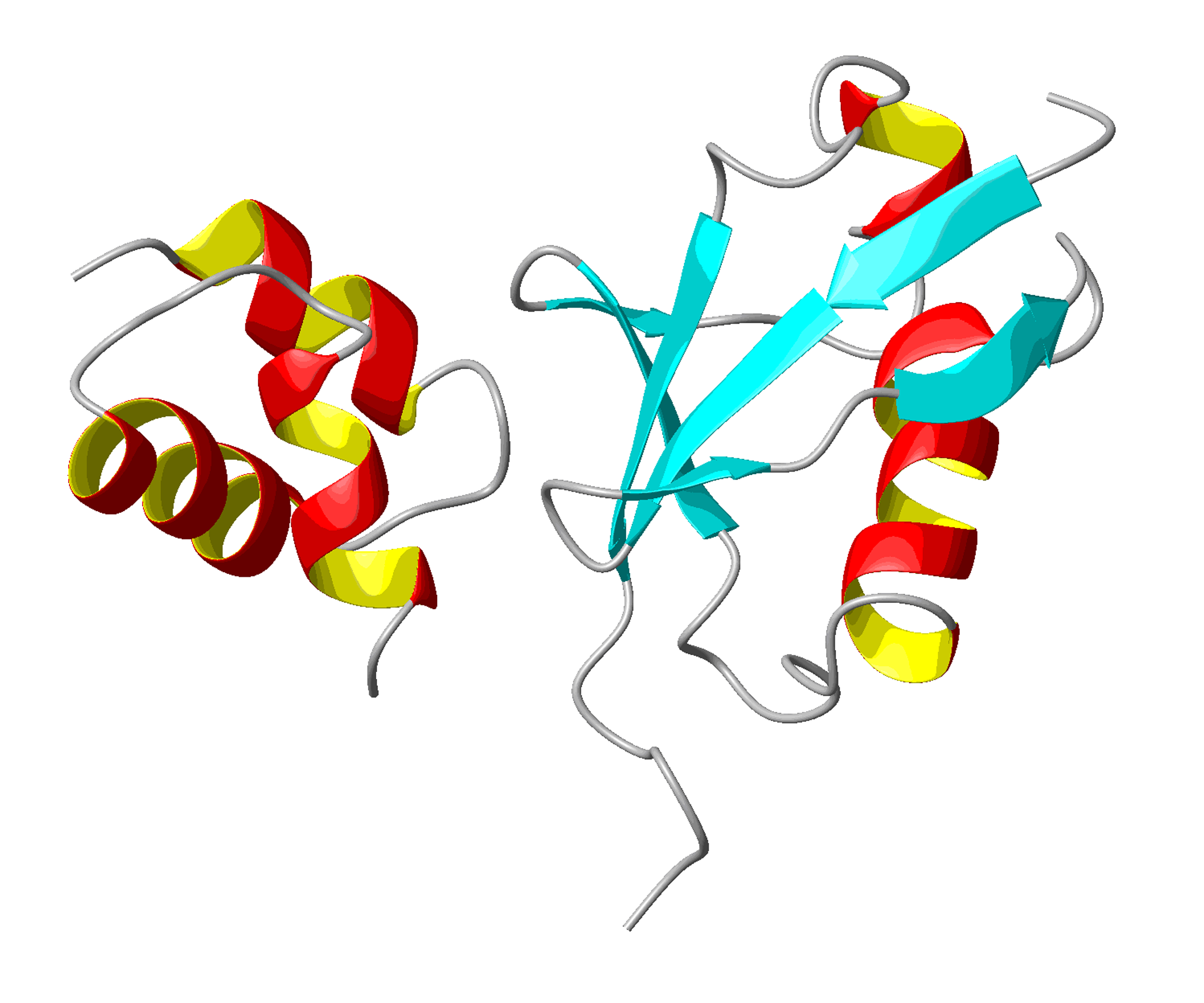

Supplement: Figure S3 — Ribbon representation of the structure of UbL-UBA1 complex as constructed by using HADDOCK method. The interfaces between UbL and UBA1 are shown. As known, for UBA1, the 666MGV668 loop between helix1 and helix2 contributes to the specific interaction. The residues Arg199, Gln219 and Ile221 are potentially located in the interface for the UbL domain of DC-UbP. Combined with mutagenesis, we thus characterized the residues Phe195 and Arg199 of UbL that are important to interacting with UBA1. (TIF) [file pone.0107509.s003.tif]

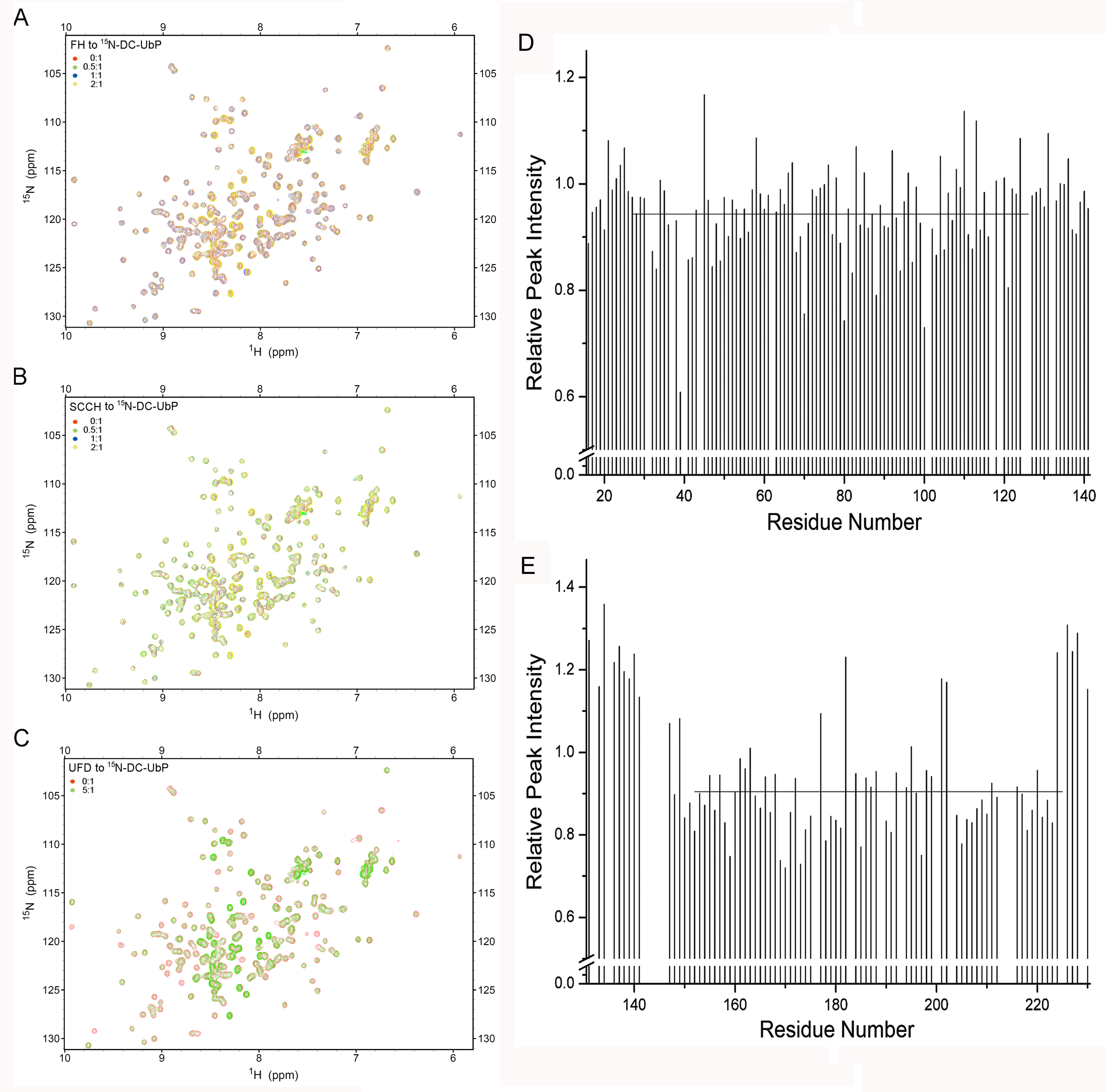

Supplement: Figure S4 — NMR titration for charactering the interactions of DC-UbP with various fragments of UbE1. A, Overlay of the HSQC spectra of 15N-labeled DC-UbP (100 µM) and addition of FH (FCCH and AD) fragment at different molar ratios. There is no considerable peak change in the spectra during titration with the FH fragment. B, As in (A), SCCH titration. There is no considerable peak change in the spectra during titration with the SCCH fragment. C, As in (A), GST-UFD titration, except that the concentration of 15N-labeled DC-UbP was 20 µM. With addition of GST-UFD, some peaks in the C-terminal part (UbL) of DC-UbP become weak or disappearing, indicating that UFD specifically binds to the UbL domain. D, Diagram of the peak intensity (height) changes of UbP_N titrated with GST-UFD at a molar ratio of 1∶3 against residue number. E, As in (D), diagram of UbP_C titrated with GST-UFD at a molar ratio of 1∶3. The lines indicate the mean peak intensities for the UBD (a.a. 27–126) and UbL (152–225) domains, respectively, suggesting that UFD specifically binds with UbL but not with UBD. (TIF) [file pone.0107509.s004.tif]

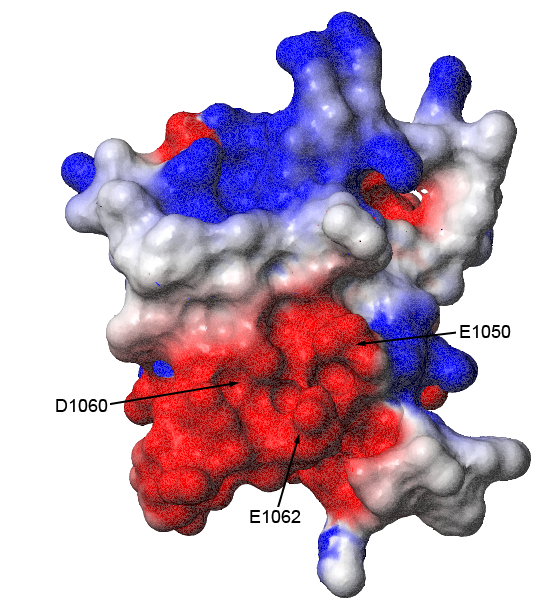

Supplement: Figure S5 — Structural model of the UFD domain of human UbE1 showing the electrostatic surface. The acidic residues on the surface corresponding to those in yeast Uba1 potentially binding to the positively-charged interface of Ubc1 (UbE2) are highlighted. The structure was generated by homology modeling using I-TASSER server and the electrostatic surface was displayed with MOLMOL. (TIF) [file pone.0107509.s005.tif]

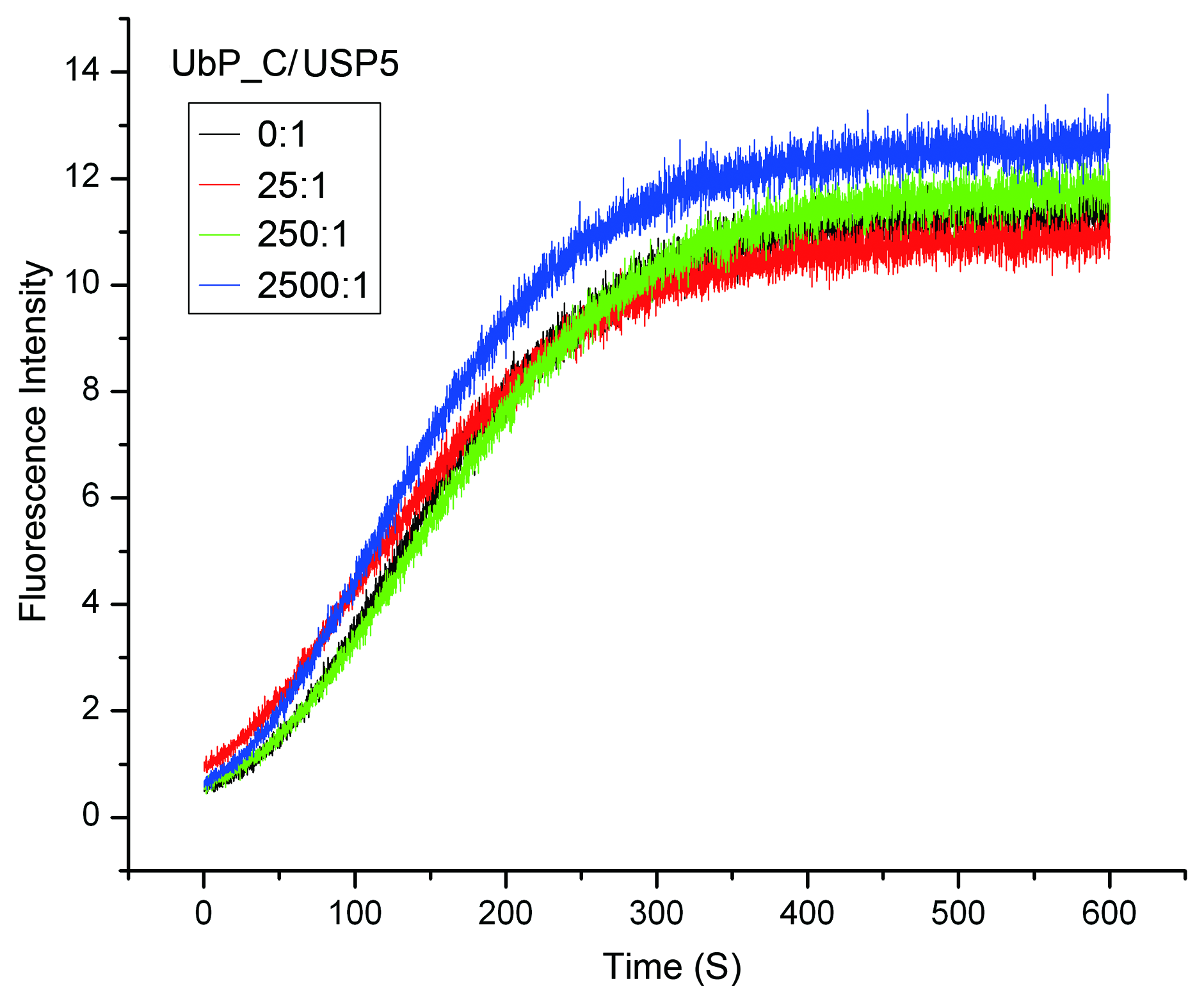

Supplement: Figure S6 — Effect of the C-terminal part of DC-UbP (UbP_C) on the deubiquitinating activity of USP5. The fluorescence increases were monitored for the deubiquitinating activities of purified USP5 in the presence of different molar ratios of UbP_C/USP5. The concentrations of USP5 and Ub-AMC were 10 nM and 250 nM, respectively. (TIF) [file pone.0107509.s006.tif]
